# Lower limb post-traumatic osteomyelitis: a systematic review of clinical outcomes

**DOI:** 10.1007/s00590-022-03364-2

**Published:** 2022-08-20

**Authors:** Paul Rodham, Michalis Panteli, James S. H. Vun, Paul Harwood, Peter V. Giannoudis

**Affiliations:** 1 NIHR Academic Clinical Fellow, Academic Department of Trauma & Orthopaedics, School of Medicine, Leeds General Infirmary, University of Leeds, Clarendon Wing, Level D, Great George Street, Leeds, LS1 3EX UK; 2 Lecturer in Trauma & Orthopaedic Surgery, Academic Department of Trauma & Orthopaedics, School of Medicine, University of Leeds, Leeds, UK; 3 Higher Surgical Trainee in Trauma and Orthopaedics, Academic Department of Trauma and Orthopaedics, Leeds Teaching Hospitals NHS Trust, Leeds, UK; 4 Consultant Trauma and Orthopaedic Surgeon, Academic Department of Trauma and Orthopaedics, Leeds Teaching Hospitals NHS Trust, Leeds, UK; 5 Professor of Trauma and Orthopaedics, Academic Department of Trauma & Orthopaedics, School of Medicine, University of Leeds, Leeds, UK

**Keywords:** Infection, Osteomyelitis, Outcomes

## Abstract

**Purpose:**

The aim of this study was to examine the patient-reported outcomes of patients presenting with post-traumatic osteomyelitis (PTOM) of the lower limb over the past 15 years. This period was chosen to reflect modern treatment principles and increased centralisation of care.

**Methods:**

An electronic literature search of the relevant databases (PubMed, Ovid Medline, Embase, and the Cochrane library) was conducted to identify studies published between January 2006 and July 2021 reporting series of greater than 10 patients with PTOM of the tibia or femur at the site of a previous fracture. Studies reporting septic non-union were excluded.

**Results:**

Sixteen eligible studies were identified and included in the final report. Remission of infection was achieved in 93.2% of cases (range 70–100%), whilst amputation was reported in 1–7% of cases. A variety of patient-reported outcome measures were utilised including the lower extremity functional scale, short musculoskeletal functional assessment, Enneking score, and EQ-5D-3L. Limb-specific functional outcomes returned to levels similar to that of the general population although poorer outcomes were noted in specific cohorts including those with complex anatomic disease and active medical comorbidities.

**Conclusion:**

Infection following fracture fixation remains a difficult problem to treat. Regardless, using modern treatments and techniques patients can have comparable functional outcomes to that of the general population. High-quality studies are required to advance our knowledge into which types of treatments offer a benefit and how to further improve outcomes.

## Introduction

Osteomyelitis is an infectious disease that affects the bone and bone marrow [1]. In adults, it most commonly occurs following trauma as a result of inoculation with bacteria such as *Staphylococcus aureus.* The causative organisms are particularly virulent due to their ability to form local microcolonies whereby they can escape detection and clearance by cells of the innate immune system. Particularly in the presence of foreign material, it is able to secrete a physical barrier of polysaccharide and proteinaceous material that results in the formation of a biofilm, further shielding microorganisms from immune cell detection [2]. Furthermore, *Staphylococcus Species* are able to penetrate and reside deep within the bone canalicular system where they can reside for years at a time, potentially explaining the reason for late reactivation of previously “cured” infections [3].

The presenting features of post-traumatic osteomyelitis are often non-specific, however may include pain, swelling and erythema at the site of a previous fracture, new onset wound drainage, radiological features such as implant loosening, sequestra formation or failure of healing; or a systemic inflammatory response associated with pyrexia and raised inflammatory markers [4]. A recent consensus definition established that fracture-related infection is diagnosed when any of the following criteria are met: (1) fistula, sinus, or wound breakdown in communication with bone of an implant; (2) purulent wound drainage or the presence of pus during surgery; (3) pathogens isolated from at least two separate deep tissue samples; or (4) presence of microorganisms in deep samples as confirmed by histopathological staining techniques [4].

Early classifications utilised a temporal classification with fracture-related infections classified into early (< 2 weeks), delayed (2–10 weeks), or late (10 weeks) [5]. This was subsequently built upon by the Cierny–Mader classification which aimed to describe both the anatomy of the disease and the fitness of the host to reflect the varying complexity of this condition [6]. Most recently the BACH classification has been demonstrated to correlate well with clinical outcomes, more accurately representing the heterogenous nature of osteomyelitis through descriptions of the bone involvement, antibiotic options, requirement for soft tissue reconstruction, and degree of optimisation of the host for surgery [7].

The principles of treating post-traumatic osteomyelitis vary according to the duration of infection, and the condition of the host. In the acute setting where fracture healing has not yet taken place a decision is often made to retain metalwork, seeking to suppress the infection sufficiently with debridement and antibiotics until osseous union is achieved, before metalwork removal [8]. In the chronic setting where the infection is well established, treatment principles include the removal of all foreign material present, debridement of all devitalised bone and soft tissue, provision of osseous stability, management of the dead space, reconstruction of the soft tissue envelope if required, and local and systemic antibiotic therapy.

Despite advances in the basic science and treatment strategies, the condition continues to produce significant morbidity, at a high cost to healthcare providers [9]. Outcomes do appear to improve with the treatment of patients within specialist bone infection units, although these centres remain relatively novel, and long-term data is not yet available [10]. Many of the data regarding outcomes following post-traumatic osteomyelitis utilise disease remission as an end-point, which whilst valuable, can often fail to fully represent the outcomes that are important to patients in terms of limb function, walking ability, and return to work [11]. Due to the rarity of this condition and previous lack of centralisation of care, many of these studies are often small, often containing fewer than 20 cases.

Increasingly within Orthopaedics, the use of patient-reported outcome measures (PROMs) is becoming widespread and better represents those outcomes that are important to patients [12]. Through the use of PROMs, we can better counsel patients to the spectrum of potential outcomes they can expect to achieve, whilst also increasing the value of clinical studies assessing current and novel treatment modalities [13]. Although associated with higher costs due to time and resource requirement, the collection and utilisation of PROMs are likely to become commonplace within modern Orthopaedic practice [13].

The aim of the current study was to assess the clinical outcomes and where available the PROMs of patients with post-traumatic osteomyelitis of the lower limb over the past 15 years, a period which more accurately represents modern treatment strategies with increasing centralisation of care of these patients.

## Methods

A systematic review was conducted in accordance with the guidance described in the Cochrane handbook for systematic reviews [14], and presented in accordance with PRISMA guidelines [15].

The primary outcome of the presented review was to report rates of infection remission in patients undergoing surgical management for PTOM. Secondary outcomes included PROMs, clinical findings, and amputation rates.

**Fig. 1:**
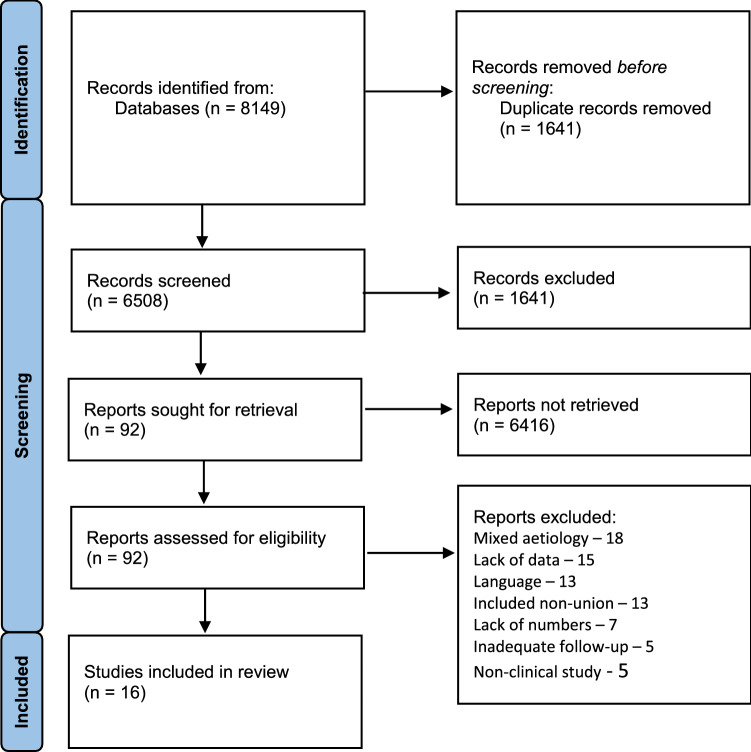
Flow chart of the study selection

A search of the relevant electronic databases was conducted (PubMed, Ovid Medline, Embase, and the Cochrane library) in November 2021 to retrieve all relevant articles using the keywords “osteomyelitis” and “trauma”, by two authors (PR and MP), in an independent, unbiased manner. In case of disagreement, inclusion of a study was decided by consensus. Bibliographies of all identified relevant articles including reviews were searched for potentially relevant articles. The flow chart of study selection is presented in Fig. 1.

Inclusion criteria for the selection of studies included: (1) studies reporting on the outcomes of patients with chronic osteomyelitis at the site of a previous tibial or femoral fracture; (2) inclusion more than 10 patients; (3) the article was published between January 2006 and July 2021; (4) the full text article was available in the English language. Exclusion criteria involved: (1) studies reporting on osteomyelitis without a previous history of trauma; (2) studies reporting on osteomyelitis in the paediatric population (before skeletal maturity); (3) studies reporting on acute osteomyelitis; (4) studies reporting on septic non-union; (5) studies where inadequate data were made available.

Each citation was reviewed for eligibility. Citations were initially reviewed on the basis of title and abstract. The remaining manuscripts were obtained and reviewed. Relevant information including authorship, publication year, study type, case number and anatomic site, surgical management, antibiotic management, duration of follow-up, infection remission rate, and other available outcome data were recorded. Qualitative results were summarised and presented in tables. Statistical comparison was not made between studies, due to the heterogeneity in methodological approach.

## Results

The initial search strategy identified 6508 citations once duplicates were removed. Following exclusions based upon information provided within the title and abstract, 92 full texts were obtained and reviewed. Sixteen papers met the inclusion criteria and were therefore included [7, 11, 16–29]. The reasons for exclusion of the remaining 76 are summarised in Fig. 1. The citations reviewed for the study included 11 retrospective case series [11, 16–19, 23–28], two retrospective comparative studies [22, 29] and three prospective case series [7, 20, 21].

Across the 16 studies a total of 664 patients including 531 patients with PTOM of the tibia, and 133 patients with PTOM of the femur were analysed. The population was 77% male with ages ranging from 16 to 88 years. Management was single stage in six studies (309 patients) [7, 19–21, 26, 28], two stages in six (165 patients) [18, 22–25, 27], and in four studies (190 patients) [11, 16, 17, 29] the decision for staged management was left to the operating surgeon. All patients underwent an initial debridement followed by a surgical strategy aiming to eliminate dead space, provide stability and appropriately cover the soft tissues where required. This was subsequently followed by systemic antibiotic therapy. Twelve studies (412 patients) [7, 11, 16, 17, 20–25, 27, 29] supplied local antibiotics through a range of techniques including antibiotic nails, antibiotic cement spacers, and antibiotic-impregnated beads. Fixation was required for 303/461 (66%) patients in the 12 studies [11, 16, 17, 19–25, 27, 29] that commented on this. Soft tissue reconstruction was required in 338/664 (51%) of cases. Duration of follow-up averaged between 12 and 50 months. A summary of the treatment strategies is provided in Table 1, whilst a summary of outcomes is provided in Table 2.

**Table 1:** Treatment strategies in patients presenting with PTOM

Author	Year	Site (*n*)	Surgical management				Systemic antibiotics
			Number of stages (*n*)	Local antibiotic provision (*n*)	Method of fixation (*n*)	Soft tissue coverage (*n*)	
Campbell et al. [11]	2011	Tibia (12)	Single stage (2) Two stages (10)	Commented on but not specified	Circular Ex-fix (8) Monolateral Ex-fix (4)	Free flap (4) Pedicled flap (4) Fasciocutaneous (4)	Average 12.8 months (1.8–33). Regimen not specified
Hong et al. [17]	2017	Tibia (103) 17 additional sites	Single stage (69) Two stages (51)	Antibiotic beads (25)	External fixation (52)	Perforator only flap (77) ALT plus pedicled flap (44)	Provided for 4–6 weeks post-operatively, regimen not specified
Jihui et al. [18]	2018	Tibia (16) Femur (2)	All two stages	None	Not specified	Lat Dorsi myocutaneous flap in all patients	Regiment not specified
Khan et al. [19]	2012	Tibia (20)	All single stage	None	Ex-Fix (14) Plate (6)	Radial forearm flap in all patients	Not specified
McNally et al. [20]	2016	Tibia (41) (3 with talus also) Femur (24) Tibia and Femur (1)	All single stage	Cerament G—Gentamicin eluting calcium sulphate + hydroxyapatite	External fixation (13) IMN (2)	Local flap (5) Free flap (15)	Empirical glycopeptide + carbapenem for between 6 and 12 weeks depending on culture
Qin et al. [29]	2018	Tibia (42) Femur (22)	Single or two stages depending on surgeon assessment. Numbers not reported	Experimental group received antibiotic-loaded calcium sulphate (54)	External fixation used in all cases	Not required	Intravenous antibiotics for 14–32 days. Regimen not specified
Xu-sheng et al. [22]	2017	Tibia (40)	All two stages	Antibiotic beads (18) Antibiotic spacer (22)	External fixation (27) Internal fixation (6)	Required in 18 pts, type not specified	Intravenous antibiotics for an average of 16 days. Regimen not specified
Reilly et al. [23]	2016	Tibia (41)	All two stages	Antibiotic-impregnated cement nail	Antibiotic nail only	Not required	IV antibiotics for a minimum 6 weeks directed by infectious disease service
Wu et al. [24]	2017	Tibia (16) Femur (19)	All two stages	Antibiotic-impregnated PMMA spacer	External fixation (9) Internal fixation (plate/IMN) (26)	Free flap (5) Local rotation flap (3)	Provided for average 2 weeks (range 1–6 weeks). Regimen not specified
Yu et al. [25]	2017	Femur (13)	All two stages	Antibiotic-impregnated PMMA spacer	Antibiotic coated locking compression plate used in all cases	Not required	Provided for a minimum of 4 weeks. Regimen not specified
Egol et al. [26]	2009	Tibia (24) Femur (16)	All single stage	None	Not reported	Required in 7—not specified	Not specified
Li et al. [27]	2019	Tibia (18)	All two stages	Antibiotic-impregnated PMMA spacer	External fixation used in all cases	Local pedicled flap in all cases	Not specified
Kendall et al. [28]	2019	Tibia (88) Femur (1)	All single stage	Not specified	Not specified	Free gracilis flap (80) Free lat dorsi flap (11) Rectus abdominis flap (1) Vastus lateralis flap (1) Fibula flap (1) Lateral thigh flap (1)	Provided from 6 to 12 weeks, initially broad spectrum then targeted once samples available
Hotchen et al. [7]	2020	Tibia (34) Femur (22) Also included 15 other site	All single stage	Calcium sulphate beads or calcium sulphate + hydroxyapatite (both with gentamicin). None for segmental defects	At the discretion of the operating surgeon—not specified	Not specified	Oral or IV antibiotics prescribed by a specialist musculoskeletal infectious disease physician
Qin et al. [21]	2018	Tibia (35)	All single stage	Antibiotic-impregnated calcium sulphate	External fixation used in all cases	Not required	Provided for a minimum 6 weeks. Initially broad spectrum, targeted once samples available
Arshad et al. [16]	2021	Femur (14) (11 post-trauma)	Not specified	Calcium sulphate beads impregnated with vancomycin + gentamicin	External fixation (4) Intramedullary device (2)	Not required	Minimum of 6 weeks IV

Ex-Fix—External fixator, ALT—Anterolateral Thigh, Lat dorsi—Latissimus Dorsi, IMN—Intramedullary Nail, PMMA—Polymethyl Methacrylate

**Table 2:** Outcomes of patients presenting with PTOM

Author	Year	Length of follow-up (months)	Eradication of infection	Other outcomes measured
Campbell et al. [11]	2011	50 (26–72)	100%	Ambulation—12/12 Ongoing pain—2/12 Amputation—1/12 Average LEFS—51 (14–80) representing 64% (18–100%) of maximal function
Hong et al. [17]	2017	24	Primary remission (after first reconstruction)—91.6% Secondary remission (at final follow-up)—98.3%	Na
Jihui et al. [18]	2018	Not stated—minimum 12	100%	Paley’s criteria: Excellent—12 Good—5 Moderate—1
Khan et al. [19]	2012	22.5 (19–36)	100%	Ability to ambulate without crutches—achieved in all at an average of 7.9 months (5–19 months) Non-validated subjective questionnaire—all 20 happy with functional and aesthetic outcome
McNally et al. [20]	2016	19.5 (12–34)	97%	Amputation 1/66
Qin et al. [29]	2018	29	Calcium sulphate group—98% Irrigation group—70%	Na
Xu-sheng et al. [22]	2017	Bead group 30 (18–52) Spacer group 31 (18–54)	Bead group—88.9% Spacer group—90.9^	Na
Reilly et al. [23]	2016	20 (6–76)	76%	Amputation—3/41 (AKA)
Wu et al. [24]	2017	29.5 (24–45)	97%	Average LEFS—65.6 (37–80) representing 82% (46–100%) of maximal function
Yu et al. [25]	2017	17.8 (12–24)	92%	Average knee range of movement—122 degrees (100–135)
Egol et al. [26]	2009	21.1 (10–57)	86%	Amputation—1/40 (AKA) SMFA (avg for population is 50): Mean dysfunction score—53.8 (41.9–76.3) Mean bother index—51.5 (42.6–73.9)
Li et al. [27]	2019	29.7 (24–36)	94%	Enneking score: Pre-op—9.78 ± 1.26 Post-op—24.44 ± 4.27 representing 81.5% of normal function
Kendall et al. [28]	2019	42 (11—131)	91%	Amputation—1/89
Hotchen et al. [7]	2020	PROMS—12 Clinical outcomes—24	97%	EQ-VAS: Pre-operative—58.2, at 1 year—78.9 EQ-5D-3L index scores: Pre-operative 0.284, at 1 year—0.740 (similar to age-matched general population)
Qin et al. [21]	2018	33.7 (25–41)	97%	Paley’s criteria: Excellent—13 Good—15 Fair—6 Poor—1
Arshad et al. [16]	2021	21.4 (7–49)	Primary remission—43% Remission after repeated treatment—93%	Amputation—1/14 EQ-VAS (*n* = 9): 61.6 (popn norm 82.8) EQ-5D-3L index score (*n* = 9): 0.360 (popn norm 0.856)

### Clinical outcomes

Across the 16 studies [7, 11, 16–29] eradication of the infection was achieved in 93.2% of cases with a range of between 70 and 100%. Eleven studies(7,11,27,17,18,20–21,24–26) representing 465 patients reported remission rates of greater than 90% with a further two studies [22, 26] (80 patients) reporting remission rates of greater than 85%. Noteworthy, these figures were often reported at the cessation of treatment, as opposed to following the initial treatment attempt. Primary remission rates within the studies included were reported to be as low as 43%, although this figure was reported in a cohort of patients with complex osteomyelitis [16]. Qin et al. [29] reported the lowest infection remission rate in the 20 patients managed with antibiotic-laden irrigation suction as their control group. The remission rate within this group was just 70%, significantly lower than the 98% remission rate achieved in patients managed with antibiotic-laden calcium sulphate as part of their experimental arm. They also noted that the calcium sulphate group had significantly lower length of hospital stay, shorter external fixator index, and fewer docking obstructions. Xu-sheng et al. reviewed the effect of antibiotic delivery method on remission rates, noting no difference in the rate of remission when comparing antibiotic spacers with antibiotic beads [22].

Regarding clinical outcomes, a number of assessment techniques were employed. Two authors (53 patients) [18, 21] used Paley’s criteria which combines four bone healing criteria (union, infection, deformity, and leg length discrepancy) with five functional criteria (limp, ankle equinus, soft tissue dystrophy, pain, and inactivity) to describe the outcome as excellent, good, fair or poor [30]. In their series of 16 patients with tibial PTOM and 2 with femoral PTOM, Jihui et al. [18] reported an excellent result in 12, a good result in 5, and a fair result in 1. Qin et al. [21] reported 13 excellent, 15 good, six fair, and one poor result in their series of 35 patients with tibial PTOM. Whilst Paley’s criteria provide an impression of overall function, in our experience it does not necessarily reflect true functional outcomes that may be better described through the use of patient-reported outcome measures.

Two studies (32 patients) [11, 19] specifically reported on return to ambulation. Campbell et al. reported that all 12 of their patients treated for tibial osteomyelitis returned to walking, albeit with two having ongoing pain^11^. Khan et al. also reported that all of their 20 patients with tibial osteomyelitis also returned to walking at an average of 7.9 months^19^.

Six studies (265 patients) [11, 16, 20, 23, 26, 28] reported amputations within their cohort during the course of treatment. Five authors reported a single amputation (196 patients) [11, 16, 23, 26, 28], however in the study by McNally et al. [20] this was due to an ulcer caused by a poorly fitting prosthesis who was being treated for osteomyelitis of their tibial remnant. Reilly et al. [23] reported three above knee amputations in their cohort of 41 patients with tibial PTOM. Amputation rates therefore ranged from 1 to 7%. In our experience whilst amputation may be seen an indicator of a poor outcome, it’s use in selected patients was the morbidity of limb salvage will likely be high can result in an accelerated return to function that would not otherwise be achieved.

### Patient-reported outcome measures

Six studies (179 patients) [7, 11, 16, 24, 26, 27] reported on PROMS following management of PTOM. A variety of PROMS were utilised in the 6 studies including (1) Lower Extremity Functional Scale (LEFS), (2) Short Musculoskeletal Function Assessment (SMFA), (3) Enneking score, (4) EQ-5D-3L, and (5) EQ-VAS. The LEFS is a score that assesses an individual’s ability to perform 20 daily activities, each graded 0 to 4, with a higher score representing better function [31]. SMFA consists of a 46-question assessment consisting of both a dysfunction and a bother score with a higher score demonstrating increased morbidity (the average score for the general population is 50) [32]. The Enneking score is a 6-index score designed for musculoskeletal tumours assessing pain, activity, self-perception, brace application, walking ability, and gait change [33]. Finally, the EQ-5D-3L and EQ-VAS are designed to assess quality of life through assessment of five domains including mobility, self-care, ability to perform usual activities, pain/discomfort, and anxiety/depression [34]. Each domain is scored out of three: 1—no problems, 2—some/moderate problems, and 3—severe problems. The EQ-5D-3L score is then converted into an index score with weighting based on the local population that produces a quality-of-life score between 1.000 (a perfect health state) and -0.543 (a state worse than death). The EQ-VAS is a visual analogue score that asks participants to estimate their current health state on a scale of 0 (worst health they could imagine) to 100 (best health they could imagine).

Both Campbell et al. (18) and Wu et al. (40) reported utilising LEFS. Campbell et al. [11] reported on 12 patients with PTOM of the tibia demonstrating an average score of 51 (range 14–80) representing 64% of maximal function. Wu et al. [24] reported on a series of patients with Cierny–Mader anatomic type IV affecting the tibia in 16 patients and the femur in 19 patients. They demonstrated an average LEFS score of 65.6 (range 37–80) representing 82% of maximal function. Neither of these studies reported on factors that predicted LEFS.

Egol et al. [26] reported on the short musculoskeletal function assessment (SMFA) in their including 24 patients with tibial PTOM and 16 with femoral PTOM. Within their cohort, the average dysfunction score was 53.8 (range 41.9–76.3) and the main bother index was 51.5 (range 42.6–73.9) suggesting no overall difference in function when this population is compared to the general population, although the rate of chronic pain was higher than that of the general population.

Li et al. [27] employed the Enneking score in the assessment of 18 patients with tibial PTOM. Prior to treatment, their cohort had an average score of 9.78 ± 1.26 representing 32.6% of maximal limb function. This then increased to 24.44 ± 4.27 following their two-stage management representing 81.5% of maximal limb function. Just two patients within this cohort had a score < 21 following treatment.

Finally, Arshad et al. [16] and Hotchen et al. [7] reported on the EQ-5D-3L and the EQ-VAS in their respective cohorts. Arshad et al. [16] reported on the outcomes of 9 of their 14 patients with polymicrobial Cierny–Mader type 3 and 4 osteomyelitis of their femur, all treated by a single surgeon. They reported an average EQ-5D-3L index score of 0.36, and an average EQ-VAS of 61.6. Both scores were significantly below that of the UK population norms of 0.856 and 82.8, respectively. The authors did not comment on predictors of outcome, though given that they were treating patients with complex osteomyelitis their cohort likely does not reflect the “average” PTOM patient.

Hotchen et al. [7] detailed the outcomes of 40 of their 71 patients at both baseline and at one-year post-treatment. They demonstrated significant improvements of EQ-VAS from 58.2 to 78.9 at one year post-operatively, alongside significantly improved EQ-5D-3L index scores from 0.284 to 0.74 at one year post-operatively. Within this population, outcomes were significantly better in the BACH uncomplicated group (EQ-5D-3L 0.9 uncomplicated vs 0.685 complicated, EQ-VAS 87.1 uncomplicated vs 73.6 complicated). This demonstrates that patients with uncomplicated osteomyelitis are able to achieve an overall disease state that mirrors the age-matched population, whilst those with complex osteomyelitis report a lower comparative quality of life. Predictors of outcome at one-year post-surgery within the Hotchen study included patients with simpler bone lesions (cavitary lesions vs segmental lesions or lesions affecting the adjacent joint, EQ-5D-3L 0.841 vs 0.445, EQ-VAS 84 vs 56.8), the host health status (EQ-5D-3L 0.87 healthy vs 0.627 comorbid, EQ-VAS 85.6 healthy vs 71.1 comorbid), and limitations in antimicrobial options (EQ-5D-3L only, 0.606 limited group vs 0.804 good antimicrobial options group). The requirement for reconstruction of the soft tissues did not affect the outcome at one-year post-surgery. No studies made a comparison for outcome by site of surgery (femur vs tibia).

## Discussion

Studies examining the outcomes of patients with PTOM are infrequent and often suffer from methodological flaws due to their retrospective design, low numbers, inconsistent and generally short-term follow-up, and the heterogenous nature of PTOM and its treatment. As a result, it is difficult to draw robust conclusions from the current literature, and similarly it is difficult to counsel patients as to the likely outcome that they will achieve following treatment. Historically, outcomes within this patient population have often been made binary, such as disease remission and amputation, however these do not wholly represent the outcomes that patients care about. Whilst eradication of infection is an important marker of success, these patients will often still have functional deficits. Furthermore, historically amputations are regarded as a symbol of failed treatment, however data within the trauma population has demonstrated that functional outcomes can be better with primary amputation when compared to the potential morbidity of a limb salvage approach [35].

Overall recurrence rates of PTOM were low, averaging 6.8% at final follow-ups ranging from 12 to 50 months. In the majority of studies, recurrence was taken at the conclusion of treatment, which may have failed to report additional procedures required to attain control of the infection. Two studies reported on primary versus secondary remission, demonstrating primary remission rates of 43% and 91.6%, both of which improved to 93% and 98.3%, respectively, at the conclusion of treatment [16, 24]. The lower rate of remission seen by Arshad et al. may be explained by the fact that they were treating polymicrobial infection in patients who suffered from Cierny–Mader anatomic type 3 and 4 disease.

Earlier literature would suggest that most recurrences occur within the first two years following treatment. Tice et al. [36] found that 95% of recurrences occurred within one year following treatment in their cohort receiving single line antibiotic therapy via their outpatient antibiotic therapy service, also noting significantly higher recurrence rates when the responsible organism was *Pseudomonas aeruginosa.* The inclusion of patients requiring only a monotherapy may however exclude those patients with more resistant microbiological disease, whose outcomes may be worse. Similarly McNally et al. [37] reviewed a series of 759 patients with long bone infection over an average period of 43.7 months (range 12–131 months), noting that 92.4% of their 52 recurrences occurred within the first two years following treatment. They also reported that late recurrences, occurring after 3 years, tended to follow new injuries or procedures, and often had different organisms to those of the original infection.

Six studies [11, 16, 20, 23, 26, 28] reported on amputation rates which ranged from 1 to 7%, all of which were performed following failure of a limb salvage approach. Historically amputation has been utilised as a measure of assessing a poor outcome, however there is an increasing body of literature supporting the consideration of early amputation in trauma under circumstances where the outcome is likely to be poor, or the morbidity of treatment high. Particularly in those patients who are likely to require amputation, the use of early amputation is associated with fewer overall complications, more frequent prosthetic use, and equivalent functional outcomes to those who underwent limb reconstruction [35, 38]. Similarly, in the military population, early amputation has been associated with fewer psychological complications [39]. However, there are currently no studies examining the use of early versus late amputation in the management of osteomyelitis following trauma which will guide decision making and the design treatment pathways in challenging, equivocal cases. In our experience, the appropriate use of early amputation can facilitate a swift return to a good level of function, similar to that of our limb salvage patients. This option should always be considered, although when the decision to proceed to amputation is considered it should be in conjunction with the wider multi-disciplinary team including plastic surgery, psychology, and rehabilitation medicine.

Patient-reported outcomes were only reported in 38% of the studies (*n* = 6/ 16) [7, 11, 21, 24, 26, 27]. In their series of 12 and 37 patients, Campbell et al. [11] and Wu et al. [24] recorded a mean LEFS of 51 and 65.6, respectively. The lower scores reported by Campbell et al. could be explained by the higher number of procedures undertaken (mean 11.4 vs mean 5.3) and the more frequent requirement for soft tissue reconstruction, which may suggest more complex disease within their cohort. Neither study reported any significant association between disease characteristics or treatment strategy and LEFS. Within the non-injured population, the median LEFS is 77, substantially higher than that seen in both cohorts where this measure was utilised on PTOM patients [40]. Similarly, the LEFS returns to a mean of 69.4 at 24 weeks following ankle fracture [41]. This suggests that even despite disease control, the functional outcomes following fracture-related infection are lower than those seen in both the healthy and the fracture populations.

Li et al. [27] utilised the Enneking score to assess their cohort of 18 patients with tibial PTOM treated with staged debridement and vacuum seal-assisted local flap coverage. At an average of 29.7 months post-operatively, limb function was improved from 9.78 (32.6% maximal function) to 24.44 (81.5% maximal function), with only a single case of recurrence. Although well reported within musculoskeletal tumour surgery, the use of the Enneking score is uncommon in trauma surgery. It has previously been demonstrated to correlate with the American Orthopaedic Foot and Ankle Society (AOFAS) score in open ankle injuries, and is currently recommended for use by the British Association of Plastic Reconstructive and Aesthetic Surgeons (BAPRAS) standards for the management of lower limb trauma [42]. It has not been independently validated in limb reconstruction post-trauma and therefore should be interpreted cautiously [33].

In their retrospective single-surgeon series of 32 patients with lower limb osteomyelitis Egol et al. [26] utilised SMFA to assess functional outcomes. The SMFA is a well-validated functional scoring system in both general Orthopaedics and Trauma, that correlates well with the patients ability to perform their usual activities of daily living and recreational activities; and is responsive to changes in a patient’s health state [32, 43]. At an average follow-up of 21.1 months, the SMFA dysfunction score was 53.8 and the bother index was 51.5, similar to those scores seen within the general population. In addition to achieving a primary remission rate of 84%, they also reported a 5% risk of recurrence in patients without recurrence for more than a year.

Hotchen et al. [7] reported on the outcomes of 56 patients with tibial and femoral osteomyelitis which were stratified via their previously published BACH classification [44]. They were able to demonstrate a significant improvement and return to the age-matched population norm in both the EQ-5D-3L (0.284 pre-op vs 0.74 post-op) and the EQ-VAS (58.2 pre-op vs 78.9 post-op) at one-year post-surgery. Within their cohort, Hotchen et al. established that both quality-of-life indicators were significantly improved with the presence of uncomplicated disease, simpler bone lesions, and limited comorbidities. Of note, within their study the baseline scores of H2 (poorly controlled comorbidity) patients were significantly lower than those of the H1 (fit and well or well-controlled disease) patients, and whilst the 1-year outcome was significantly higher for H1 patients, the overall gain in EQ-5D-3L and EQ-VAS was equivalent between these groups.

Arshad et al. [16] reported on the outcomes of their cohort of 14 patients with polymicrobial Cierny–Mader anatomic type 3 and 4 osteomyelitis of the femur. Contrary to Hotchen et al. they reported significantly lower quality-of-life scores compared to the general population (EQ-5D-3L—0.360, EQ-VAS—61.6) at an average follow-up of 21.4 months. These lower outcome scores are likely a consequence of the more complex disease and the polymicrobial nature of the infections assessed within this study. Eleven of the 14 patients were classified as complex according to the BACH classification, which likely explains the high rate of early treatment failure (43%) within this cohort.

The strengths of this review lie within the broad search criteria, systematic methodology as per PRISMA guidelines, and the inclusion clinical indicators of outcome alongside the use of PROMS. Nonetheless, the heterogeneity of the data, the majority of which is retrospective, creates inherent flaws including selection bias, incomplete data sets, and variation in treatment strategies.

The study of infection following fracture fixation is difficult, and as demonstrated frequently incurs methodological flaws. As demonstrated in this review there is a lack of agreement as to which outcome measure should be employed [45]. The success of registries such as the national joint registry (NJR) has demonstrated the importance of “big data” in answering important clinical questions within Orthopaedics. However, the costs of establishing such registries are frequently prohibitive in conditions where the incidence is low [46]. When considering fracture-related infection, small single centre series will likely fail to provide adequate outcome data to overcome the heterogenicity of the condition and treatment techniques. Therefore, until large sample size, high-quality studies with reliable outcome measures are reported, advancement of the field may be delayed.

## Conclusion

Infection following fracture fixation remains a significant challenge within Orthopaedics. Even with modern techniques and centralisation of care, the morbidity can be substantial, particularly when complex disease is present in a morbid population. Due to the heterogenous nature of osteomyelitis and its treatment, the current literature is sparse. Hence, there remains a significant need for collaborative studies across limb reconstruction units, which will generate adequately powered, high-quality data—ultimately empowering meaningful conclusions to be made on patient’s treatment based on patient outcomes.
